# Primary Care, Public Health, and Mental Health

**Published:** 2009-12-15

**Authors:** Benjamin G. Druss, Robert A. Mays, Valerie J. Edwards, Daniel P. Chapman

**Affiliations:** Rollins School of Public Health, Emory University; National Institutes of Mental Health, Bethesda, Maryland; Centers for Disease Control and Prevention, Atlanta, Georgia; Centers for Disease Control and Prevention, Atlanta, Georgia

Primary care providers (PCPs) can perform a central role in bridging mental health and public health. This role was recognized by the Mental Health/Mental Illness Expert Panel convened by the Centers for Disease Control and Prevention's Division of Adult and Community Health. PCPs, who form the backbone of the US health workforce, can serve a critical role in all stages of mental health delivery, from prevention through early detection, and effective ongoing care.

PCPs (in their practices) have a unique opportunity to reduce the risk for onset of mental disorders. Approximately 70% of Americans see a general practitioner in any given year (1), making these providers well placed for population-based prevention efforts. These physicians can identify psychosocial stressors and early warning signs such as insomnia, subthreshold anxiety, and depressive symptoms. Through the early identification and mitigation of these risk factors, PCPs can address these issues before they develop into diagnosable mental disorders.

PCPs are ideally poised to provide effective secondary prevention through the early detection and treatment of common mental disorders. Diagnostic tools designed for the primary care setting have been developed to facilitate the identification of common mental disorders in primary care. One example is the Primary Care Evaluation of Mental Disorders diagnostic system (2), which features an office-based system of triage and diagnosis that promotes correct identification of mental disorders. The *Diagnostic and Statistical Manual of Mental Disorders, Fourth Edition, Primary Care Version *(DSM-IV PC) includes tools for differential diagnosis of psychiatric disorders in general medical settings (3).

Primary care clinics are increasingly becoming critical sites for tertiary prevention, the ongoing treatment of common mental disorders to reduce disease. During the past 20 years, the locus of mental health treatment in the United States has shifted from specialty mental health to primary care medical settings, and more than half of treatment for mental disorders now occurs in general medical settings (4). However, time constraints, competing patient demands, and financial disincentives to treat mental disorders may make it difficult for PCPs to provide high-quality care for common mental disorders. Organized models of care, involving multidisciplinary teams, care managers, and information technology supports, can provide the resources primary care physicians need to ensure coordination and continuity of care (5).

Policy initiatives such as the patient-centered medical home movement (6) are working to create a financial and regulatory environment that can better support these organized delivery models for improving chronic disease management in the United States (6). Given the large and growing rates of psychiatric treatment in primary care (4), mental health will need to be explicitly included in these reforms. If successful, these new models will lay a foundation for integrating a public health approach and mental health while further strengthening the nation's primary care infrastructure.

## References

[1] Ezzati-Rice TM, Rohde F (2008). Variation in ambulatory health care visits and visits for general checkup by demographic characteristics and insurance status, US civilian noninstitutionalized population ages 18-64, 2005. Statistical Brief no. 201.

[2] Johnson JG, Spitzer RL, Williams JB, Kroenke K, Linzer M, Brody D (1995). Psychiatric comorbidity, health status, and functional impairment associated with alcohol abuse and dependence in primary care patients: findings of the PRIME MD-1000 study. J Consult Clin Psychol.

[3] American Psychiatric Association (1995). Diagnostic and statistical manual of mental disorders, fourth edition, primary care version.

[4] Kessler RC, Demler O, Frank RG, Olfson M, Pincus HA, Walters EE (2005). Prevalence and treatment of mental disorders, 1990 to 2003. N Engl J Med.

[5] Gilbody S, Whitty P, Grimshaw J, Thomas R (2003). Educational and organizational interventions to improve the management of depression in primary care: a systematic review. JAMA.

[6] Rittenhouse DR, Shortell SM (2009). The patient-centered medical home: will it stand the test of health reform?. JAMA.

